# A new infodemiological approach through Google Trends: longitudinal analysis of COVID-19 scientific and infodemic names in Italy

**DOI:** 10.1186/s12874-022-01523-x

**Published:** 2022-01-30

**Authors:** Alessandro Rovetta, Lucia Castaldo

**Affiliations:** R&C Research, Research & Disclosure, Brescia, Italy

## Abstract

The scientific community has classified COVID-19 as the worst pandemic in human history. The damage caused by the new disease was direct (e.g., deaths) and indirect (e.g., closure of economic activities). Within the latter category, we find infodemic phenomena such as the adoption of generic and stigmatizing names used to identify COVID-19 and the related novel coronavirus 2019 variants. These monikers have fostered the spread of health disinformation and misinformation and fomented racism and segregation towards the Chinese population. In this regard, we present a comprehensive infodemiological picture of Italy from the epidemic outbreak in December 2019 until September 2021. In particular, we propose a new procedure to examine in detail the web interest of users in scientific and infodemic monikers linked to the identification of COVID-19. To do this, we exploited the online tool Google Trends. Our findings reveal the widespread use of multiple COVID-19-related names not considered in the previous literature, as well as a persistent trend in the adoption of stigmatizing and generic terms. Inappropriate names for cataloging novel coronavirus 2019 variants of concern have even been adopted by national health agencies. Furthermore, we also showed that early denominations influenced user behavior for a long time and were difficult to replace. For these reasons, we suggest that the assignments of scientific names to new diseases are more timely and advise against mass media and international health authorities using terms linked to the geographical origin of the novel coronavirus 2019 variants.

**Supplementary Information:**

The online version contains supplementary material available at 10.1186/s12874-022-01523-x.

## Introduction

### Background

Naming a new human infectious disease is a relevant and complex public health issue [1]. As of October 2021, health authorities and researchers have classified two principal infodemic phenomena due to the use of improper disease names: i) stigmatization, which is the association of the disease’s characteristics to a specific ethnic group or social category [2], and ii) misidentification, which is the attribution of properties of other known diseases to the new disease [3]. These caused unmotivated fears, racism, misleading information, and fake news. Although mass media and controversial public figures have often caused the spread of flawed denominations [4], some terms coined or widely adopted by the scientific community have been sharply criticized in this regard. Among the most striking examples, we find "Middle East respiratory syndrome" (MERS), which creates a direct geographical relationship between the new syndrome and the Middle East, and "Swine flu," which associates novel influenza with the swine breed [2]. In particular, the latter contributed to the severe economic damage to farmers, notwithstanding the absence of evidence of transmission through pork consumption. Besides this, the ordinary "novel [pathogen name]" formulation is limited in time and destined to generate dangerous confounding [5]. Since the advent of social networks has made the infodemic even more complex to administer [6], more attention must be paid to choosing a name. Despite such a scenario and past attempts to create standardized naming procedures for new infectious diseases, the scientific name of the illness condition resulting from the novel coronavirus 2019 infection (COVID-19) was introduced more than a month after the Wuhan outbreak (late December 2019 vs. 11 February 2020) [7]. As shown in previous papers, the first scientific acronym, "2019-nCoV," was ignored by users and media, which preferred generic and even stigmatizing names like "Chinese virus" [3, 4]. This has increased the racism towards the Chinese population [2, 3, 8], leading to the birth of the anti-discrimination campaign *#IAmNotAVirus* [9]. Moreover, the generic name "coronavirus" (in some cases "corona virus") has given rise to infodemic episodes such as the association with the Mexican beer brand "Corona" and the consultation of studies concerning previous coronaviruses [2, 4]. While some of these connections might have been jokes, their resonance is relevant since conspiracies have roots in confirmation bias, and fear can lower rational faculties [10, 11]. Indeed, some users have looked for causal correlations between Corona beer and the novel coronavirus 2019 [12]. For all these reasons, many scientists have spoken out openly, calling for the selection of harmless terms [13]. About a year after the pandemic outbreak, the infodemiological situation has further worsened due to the arrival of the COVID-19 variants of concern (VOC) [14]: once again, the delay in the assignation of official names has allowed the spread of stigmatizing terms such as - in chronological order - English variant, (South) African variant, Brazilian variant, and Indian variant [15, 16]. Unfortunately, these monikers have also been adopted by some national health authorities [16].

### Recent epidemiological and public health global scenario

COVID-19 proved to be a hazardous and devious disease. During 2020, its infection fatality rate was around 0.7% [17], ranging from 2.9% to 40% in the age groups from 65 to 95 years [18]. Numerous comorbidities and local conditions affect the severity of its course [19]. The World Health Organization (WHO) also estimates that the number of deaths due to COVID-19 until December 2020 was much higher than the official figures (i.e., over 3 million against the official 1.8 million) [^20^]. Consequently, hospitals worldwide have been overloaded with COVID-19 patients, leading to a shortage of hospitalization capacity and healthcare [21]. The sum of all these factors led to an even more devastating impact on low-and-middle-income countries (LMICs). In fact, due to limited internal resources, these nations lack adequate disease surveillance systems, health facilities, and doctors [22–24]. It is also not uncommon for the population to live in poor sanitation, which considerably increases the risk of comorbidities [25]. Finally, popular beliefs, health ignorance, and fake news create favorable conditions for the uncontrolled spread [22] and emergence of new VOCs [24]. On this point, the authors of this paper stress the importance of achieving vaccination equity and denounce the hypocrisy of stigmatization [26]. Further indirect damage from COVID-19 relates to the rise of other infectious diseases. Some notable examples are tuberculosis (e.g., Peru) [27], Zika and Mucormycosis - also known as black fungus - (e.g., India) [28], and hepatitis (e.g., Egypt) [29]. Again, reduced diagnosis and management capacity towards these illnesses has put further burdens on the already compromised health systems and created a vicious circle of mutual comorbidities between COVID-19 and other infectious diseases.

### Methodological framework and research objectives

In this complex context, Italy - one of the countries hit hardest by COVID-19 - has also faced a pressing and widespread infodemic [30]. As stated by the WHO, dismisinformation is one of the main risk factors for COVID-19 as it can lead to the assumption of harmful behaviors, non-compliance with anti-epidemic regulations, and stigmatization [31]. The purpose of this paper is multiple as it poses the following main research objectives: O1) quantify the adoption rate of scientific and infodemic (generic and stigmatizing) COVID-19 names over time in Italy, O2) quantify the adoption rate of scientific and infodemic (generic and stigmatizing) VOC names over time in Italy. To achieve our goal, we used Google Trends [32], an online tool developed by Google that allows the user to monitor netizens’ web interests in specific keywords, queries, and topics. The results are provided as relative search volumes (RSVs) ranging from 0 (minimum interest) to 100 (maximum interest). Google Trends has been successfully used in several infodemiological and even epidemiological studies. For example, Sheth et al. recently found a causal correlation between heart failure-related RSVs and heart failure mortality in the United States [33]. Flanagan et al. investigated worldwide users’ online interest in irritable bowel syndrome, concluding that Google Trends can complement traditional epidemiological methods in gastrointestinal disease [34]. Zitting et al. showed that Google Trends could provide precious information on insomnia and sleep disorders during dramatic events like COVID-19 [35]. In this regard, Springer et al. found a significant increase in publications on Google Trends during COVID-19 [36]. This is likely because Google Trends requires an intuitive approach and, compared to other more specific methods such as sentiment analysis via deep learning, its implementation is faster, and the outcomes are available without latency [37]. However, RSVs need to be interpreted cautiously and examined through appropriate methodologies [37, 38]: in addition to the mass and social media influence described above, RSVs are subject to random fluctuations and anomalies. Hence, this paper also has two secondary purposes. The first is to provide a new procedure to improve the sensitivity and accuracy of Google Trends datasets. The second is to introduce a new keyword collection methodology to identify COVID-19-related queries. Therefore, the paper is structured as follows: in the Results section, we discuss trends and relative ratios of scientific and infodemic terms related to COVID-19 and its variants. The aim is to provide a temporal and spatial (i.e., national and regional) picture of the distribution of the various monikers. Since the authors of this paper are sensitive to the issue of publication bias [39], we have created a subsection for negative results as well. After that, we discuss the findings in the light of recent evidence from similar literature, contextualizing them in the current scientific scenario. Finally, we detail the methodology and statistical tests used in the paper. Here, through appropriate examples and a mathematical formulation, we present the new procedures to increase the reliability and accuracy of the datasets extracted from Google Trends. To the best of our knowledge, this is the first study to investigate such a wide range of keywords related to COVID-19, providing a way to quantify even the lowest RSVs.

## Results

### National web interest in COVID-19 names

The adoption of generic or stigmatizing keywords to identify the novel coronavirus 2019 was a frequent phenomenon in Italy during the early stages of the pandemic (Table 1). All degrees of freedom were greater than 90.5 (i.e., t ~ z).

**Table 1:** Top generic and stigmatizing COVID-19 monikers. The third column shows the weekly relative RSV peaks in January—February—March 2020 (period 1). The last column shows the percentage ratios between the absolute peak of each keyword and the peak of the keyword with the highest absolute peak

Name	Keyword	Period 1 Max	Ratios (%)
K1	coronavirus ^c^	14—87—100	100—100—100
K2	corona ^a^	13—100—71	7.8—6.8—4.0
K3	virus ^b^	72—100—97	23—4.7—4.0
K4	corona virus	18—100—88	18—15—12
K5	virus cina + virus cinese + virus wuhan ^b^	100—26—19	15—0.5—0.3
K6	coronavirus cina + coronavirus cinese + coronavirus wuhan	74—72—100	8.0—1.2—1.5
K7	corona cina + corona cinese + corona wuhan^a^	62—100—93	1.3—0.3—0.3
K8	sars + sarscov + sars-cov	95—100—70	3.0—0.6—0.4
K9	malattia cina + malattia cinese + malattia wuhan	100—5—8	0.7—0.1—0.01
K10	epidemia	36—63—100	2.2—0.5—0.7
K11	pandemia	3—18—100	0.9—0.9—4.4
K12	pandemia cina + pandemia cinese + pandemia wuhan	100—36—90	0.3—0.04—0.002
K13	contagio	14—73—100	1.6—1.4—1.6
K14	influenza cinese + influenza cina + influenza wuhan	100—36—24	0.8—0.05—0.03

Translations: *Cina* China, *Cinese* Chinese, *contagio* contagion, *epidemia* epidemic, *malattia* disease, *pandemia* pandemic

^a^the term "virus" was subtracted

^b^ the term "corona" was subtracted

^c^the terms "novel" and "nuovo" were subtracted

Since the names above do not refer specifically to COVID-19, we compared the RSVs trends before and after the Wuhan official outbreak in late December 2019, finding a marked and significant increase (Table 2).

**Table 2:** Comparison of weekly RSVs of COVID-19 generic and stigmatizing names between January 2018—December 2019 and January 2020—September 2021

	**K1**	**K2**	**K3**	**K4**	**K5**	**K6**	**K7**	**K8**	**K9**	**K10**	**K11**	**K12**	**K13**	**K14**
t	6.9	4.6	5.7	4	2.9	4.8	3.9	7.7	2.1	5.6	3.9	5.7	5.5	2.9
Δ_AV_	82	1.0	2.0	110	63	2000	61	24	6.3	7.8	10	336	4.3	24
z_U_	11.9	6.8	11.2	11.7	11	11.9	8.6	12	4.1	11.4	11.7	9.8	11.3	5.2
Δ_m_	15	0.7	0.7	2.0*	2.0*	2.0*	2.0*	17	2.0*	4.0	5.00	2.0*	2.5	2.0*

*t* Welch t-test, ∆ percentage increase, *AV* average value, *m* median, *z*_*U*_ Mann–Whitney U test z score, * calculated as a percentage difference

The term "Influenza" (flu) showed strong seasonalities in the autumn-winter period during the five years preceding COVID-19 (Fig. S1). From 2015 to 2019, the RSV peaks occurred between January and February. However, from 2020 onwards, we observed two anomalous behaviors: the first was a significant increase in RSV during and after the winter season (∆ = 165*.*9*, t* = 2*.*8), while the second was a shift in the RSV peaks of the query (March and October 2020). We highlight that the February-March 2020 increase coincides with the claims made by some Italian scientists who have erroneously and dangerously compared COVID-19 to the seasonal flu. The generic term "malattia" (disease) has also shown a slight increase but with little statistical significance (Figs. S2 and S3). Finally, the adoption of COVID-19 scientific names before and during the first wave was extremely low (Table 3).

**Table 3:** Top scientific COVID-19 names. The penultimate column shows the RSV peaks in January—February—March 2020 (period 1). The last column shows the percentage ratios between the peak of each keyword and the peak of the keyword with the highest peak (weekly RSVs)

Name	Keyword	Period 1 Max	Ratios (%)
K1	coronavirus (reference)	14—87—100	100—100—100
S1	covid + covid-19 + covid19	N.D.—12—100	N.D.—1.1—6.0
S2	ncov + 2019ncov + 2019-ncov	100—67—37	0.3—0.03—< 0.001
S3	novel coronavirus + nuovo coronavirus	2—10—100	0.02—0.1—1.1
S4	sars-cov-2 + sarscov2 + "sars cov 2"	0—28—100	< 0.001—0.02—0.05

Translations: *nuovo* new

**Fig. 1:**
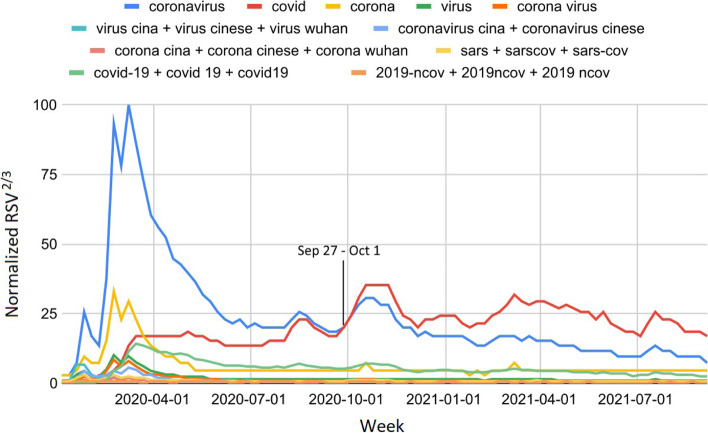
Comparison between Italian netizens’ web interest in COVID-19 scientific and top infodemic names from January 2020 to September 2021. The values shown on the y-axis are renormalized to 100

Using the RSV peak of the generic term "coronavirus" as a reference, during January 2020, the sum of the peaks (PS) of all scientific denominations reached a maximum of 0.3% (95% CI : [-1.4, 2.0]) while the stigmatizing terms’ PS reached a maximum of 26.4% (95% CI : [24.2, 28.6]). The introduction of the name "COVID-19" (February 2020) led to an improvement: indeed, scientific terms’ PS reached a maximum of 1.2% (95% CI : [-0.8, 3.2]) while stigmatizing terms’ PS reached a maximum of 2.2% (95% CI : [0.0, 4.4]). Finally, in March 2020, the scientific terms’ PS reached a maximum of 7.2% (95% CI : [5.2, 9.2]) against the stigmatizing terms’ PS equals 2.1% (95% CI : [-0.1, 4.3]). In conclusion, the generic COVID-19 names from January to March 2020 were largely the most used (total weekly RSV ≥ 91% ± 2%). A substantial portion of these was stigmatizing (up to 17% 1% during January 2020). While most keywords had the highest RSVs after the Codogno outbreak (end of February 2020) or the announcement of the national lockdown (March 9, 2020), infodemic monikers linked to the geographic origin of the virus started peaking in the second half of January 2020 (Fig. S4). The use of names to identify COVID-19 was very inhomogeneous over time (Fig. 1). In particular, the generic term "coronavirus" was by far the most adopted during the first wave. From September 2020 onwards, the more scientific name "covid" took over. The remaining infodemic denominations have had lower RSVs but persisted until nowadays. This fact is also testified by the significant increases in the medians shown in Table 1. Names like "2019-ncov" and "sars-cov-2" have been ignored by users. Moreover, in the vast majority of cases, netizens have removed the number "19" from the abbreviation "COVID-19" in their queries ("covid" vs "covid-19"-related daily RSV δ = 148.5%, t = 14.3).

### Regional web interest in COVID-19 names

**Fig. 2:**
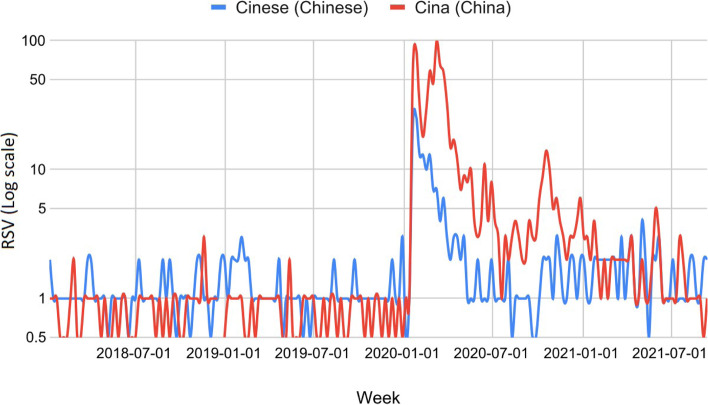
Italian netizens’ web interest in COVID-19 highly stigmatizing names from January 2018 to September 2021

**Fig. 3:**
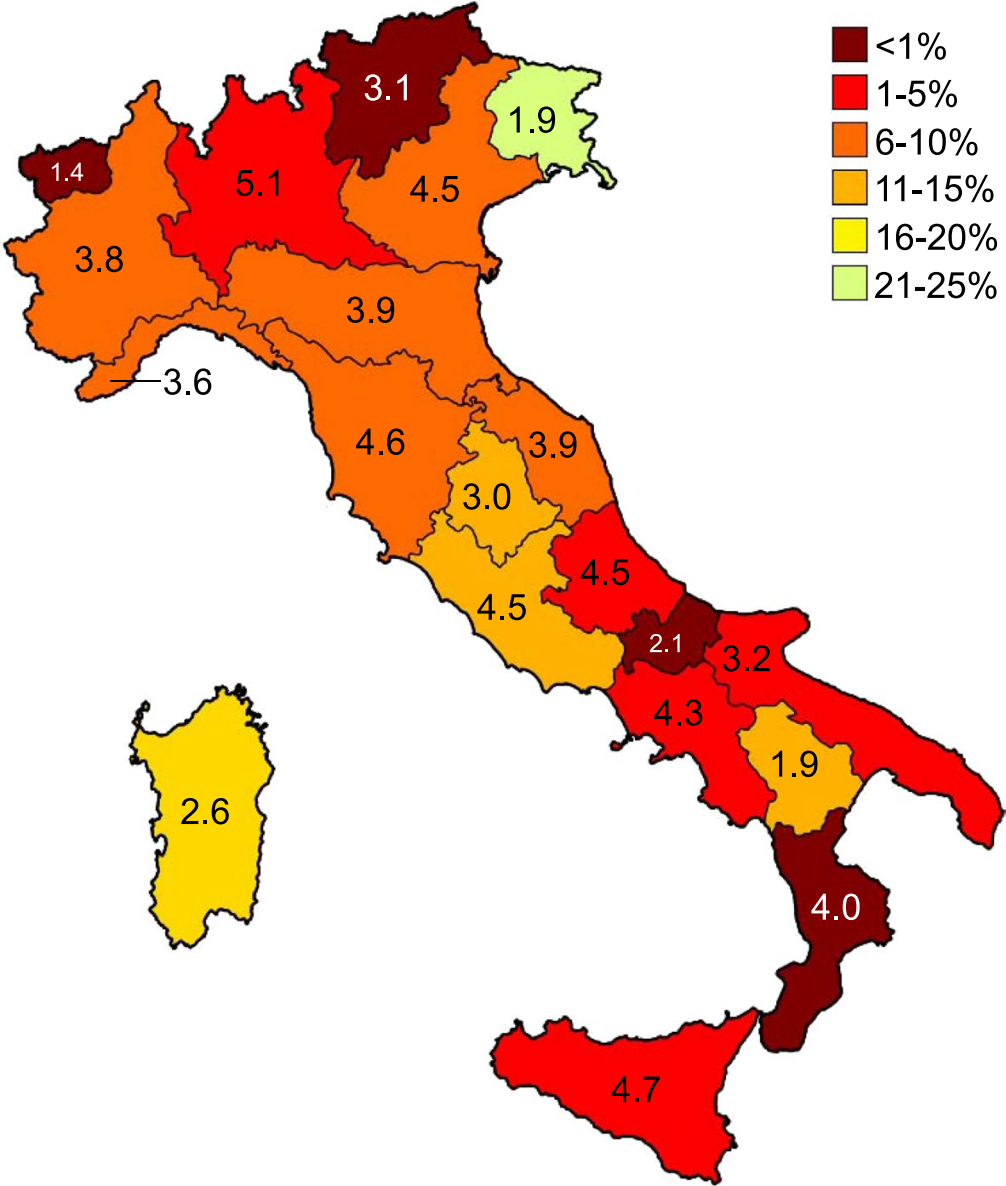
Percentage ratio between the RSV of the novel coronavirus 2019 scientific names and the RSV of the highly stigmatizing monikers in Italy before February 11, 2020. The colors indicate the ratio value, while the numbers indicate the Welch t-value

Comparing the periods January 2018 - December 2019 and January 2020 - September 2021 ("Trends over time" Google Trends section), we found that the web interest in the keyword "Cina" (China, "health" category) has significantly increased in all Italian regions (t ∈ [3.3, 5.0]). The average increase in RSV went from a minimum of 990% in Basilicata to a maximum of 2130% in Campania. The top 25 related topics and queries concerned COVID-19 in all regions. Since the last of these had a negligible RSV (<1), we concluded that the keyword was linked to COVID-19 for over 99% of the RSV. All regions showed similar behavior, i.e., three major peaks: i) end of January 2020 in correspondence with the first two cases in Italy (two Chinese tourists), ii) end of February 2020 in correspondence of the Lodi outbreak, and iii) first half of March 2020 in correspondence with the announcement of the lockdown. The only outlier is that of Friuli-Venezia Giulia, where the RSV reached its peak at the end of March 2020. A minor peak was reached during October 2020. The peaks from February to October 2020 occurred despite the official cases of COVID-19 in China being extremely low. Since all regional trends have been similar, we show the national trend of web searches (Fig. 2). As regards the queries relating to the "Chinese" keyword, the regional increases were globally significant only in the period between January and March 2020 (∆ ∈ [149, 615], t ∈ [2.1, 3.6]). Indeed, comparing the entire period January 2020 - September 2021 with the previous two years, the RSV increases in Abruzzo, Friuli Venezia Giulia, Molise, Puglia, Umbria, and Valle d’Aosta had limited significance (t ∈ [0.1, 1.2]). Through the analysis of the top queries, we excluded the keywords not related to COVID-19 and confirmed the stigma towards the Chinese population. Finally, Fig. 3 shows that - before the introduction of the term "COVID-19" (February 11, 2020) - the use of the highly stigmatizing monikers "Chinese virus" and "Chinese coronavirus" has exceeded that of scientific names like "nuovo coronavirus," "novel coronavirus," and "2019-ncov" in all Italian regions (average ratio % = 7.5 ± 1.4, CV% = 84.1).

### National web interest in COVID-19 VOC names

**Fig. 4:**
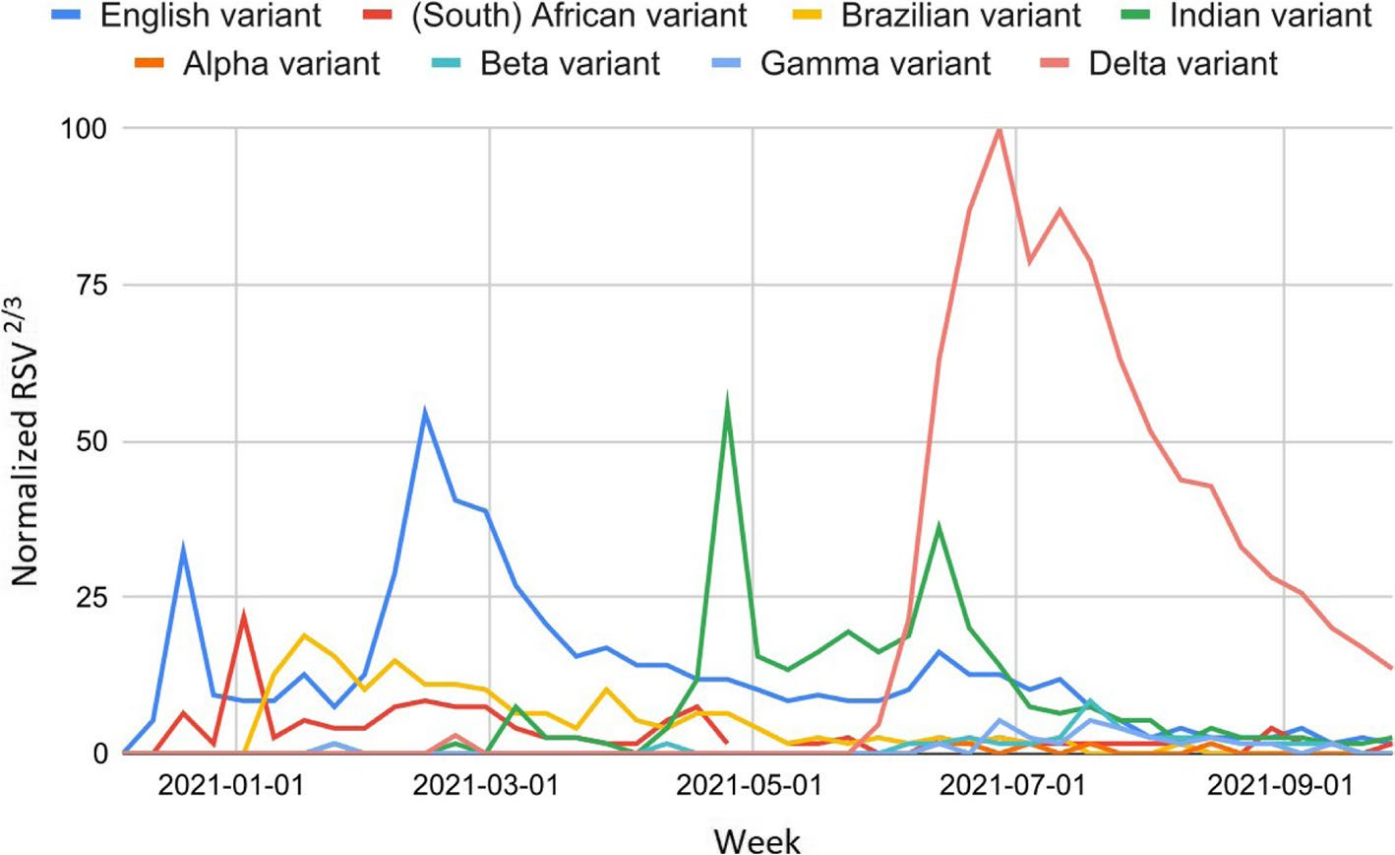
RSV over time of COVID-19 VOC-related queries in Italy from December 2020 to September 2021

Interest in COVID-19 VOCs grew rapidly with their discovery. We found extensive and widespread use of highly stigmatizing names before the introduction of the Greek letter denominations (Fig. 4). As of September 2021, the scientific names "Alpha, Beta, Gamma" did not bring benefits to the already established monikers "English variant, (South) African variant, Brazilian variant" (∆ ∈ [-99.6, -79.0], t ∈ [-4.7, -2.5]). Nevertheless, the web interest in these variants has been waning in recent months. On the contrary, the name "Delta variant" succeeded in its attempt to supplant the previous "Indian variant" (∆ = 390.3, t = 2.9). Since January 2021, the query "variante - alpha - alfa - beta - gamma - delta" (variant - alpha - beta - gamma - delta) had a substantial increase in RSV compared to the period January 2018 - December 2020 (∆ = 890.1, t = 7.5). Since the rising and top queries were all linked to COVID-19, we considered this keyword inclusive of all COVID-19 VOC generic or stigmatizing monikers. The exact list of Google Trends keywords is reported in the supplementary material (Table S1).

### Regional web interest in COVID-19 VOC names

The use of names to identify COVID-19 VOCs has been homogeneously broken down into two distinct periods: before and after the introduction of the scientific name "[Greek letter] variant" (May 31, 2021). In particular, the ratio of scientific over infodemic names ranged between 0.0 and 1.2% in all Italian regions during the first period (t ∈ [4.8, 13.5]). On the contrary, these ratios fluctuated between 232.0 and 367.4% during the second period (t ∈ [-8.5, -3.1]). However, as happened nationally, the only scientific term widely adopted was "Delta variant." The national trend, shown in Fig. 1, also well describes the regional trends (data relating to Molise and Valle d’Aosta were not available). Infodemic web searches related to COVID-19 VOCs made up 3% of web searches for the keyword "covid" from January to September 2021 (average ratio = 2.7 ± 0.1, CV% = 15.2).

### Negative results

The analysis of spatial patterns of COVID-19 names and COVID-19 VOCs names (Google Trends "subregion" section) was not possible since the datasets were compromised by a significant number of anomalies (Table S2). Specifically, 6/14 samples were incomplete (anomaly 1), while 7/14 had RSVs that strongly depended on the collection day (anomaly 3). No occasional disappearance of regions occurred (anomaly 2). Only the term "coronavirus" has proved to be sufficiently stable in time. We emphasize that this compromises the use of such a dataset to search for correlations with COVID-19 statistics. The results for the COVID-19 VOCs names were also uncertain (all samples presented anomalies 1 and 3).

## Discussion

This paper shows the overuse of infodemic names to identify the novel coronavirus 2019 in Italy. In particular, throughout January 2020, generic and stigmatizing names including "Chinese coronavirus, Wuhan coronavirus, Chinese virus, Wuhan virus" reached relevant RSV peaks. The first names introduced by the scientific community (i.e., "2019-nCoV" and "SARS-CoV-2") had an irrelevant impact, being ignored by users and, as shown in previous articles, by the mass media [4]. Health-related web searches containing the terms "China" and "Chinese" increased substantially in all Italian regions. Additionally, Google Trends’ top-related queries analysis confirmed the stigma associated with these terms. The introduction of the name "COVID-19" has mitigated the reckless use of infodemic expressions. However, until September 2020, the most used COVID-19-related word was the generic "coronavirus." This supports the hypothesis that the first names adopted to identify a new pathology have long-term effects on the population’s vocabulary. Such findings are consistent with the first impression theory [40]. In this regard, it is also necessary to consider that the Italian mass media have heavily influenced users’ web searches on COVID-19 [4]. The failure to adopt the first scientific names is plausibly linked to their complexity. Even the number "19" was truncated by the term "COVID-19" in most cases. We also point out that other generic or erroneous names, such as "epidemic" and "flu," have been widely used to identify COVID-19. Specifically, the association between seasonal flu and COVID-19 is plausibly due to the unfortunate claims of some Italian scientists and conspiracists and the resonance they have had through the press [4]. Nonetheless, scientific terms have been more used in the long run than infodemic terms. Finally, we observed that, after the first wave, the general web interest in the pandemic has steadily declined until nowadays. As noted in other papers, web interest has shifted to related and more practical topics, including smart working and learning [41, 42], drug treatments [43], vaccines [30, 44], and green-pass [45]. A similar but in some respects worse scenario was observed for COVID-19 VOCs. Indeed, stigmatizing names - linked to the geographic region in which the variant was first identified - have been commonly adopted by users, mass media, scientists, and even health agencies. The names "English variant, (South) African variant, Brazilian variant, Indian variant" were widely disseminated in all Italian regions during the December 2020 - August 2021 timelapse. The introduction of the scientific names "Alpha, Beta, Gamma" by the WHO (May 2021) was ineffective; on the contrary, the denomination "Delta" has managed to supplant its infodemic equivalent "Indian variant." Again, this provides evidence to support the first impression theory. Web interest in COVID-19 VOCs infodemic names accounted for approximately 3% of all COVID-19-related queries. However, as per COVID-19 names, scientific names have assumed a dominant trend (from "Delta" onwards). The relationships between the various regions concerning the adoption of the different COVID-19 and VOCs names have not been assessable as the Google Trends datasets proved to be very unstable. Such negative results should warn the scientific community about using Google Trends as an epidemiological surveillance tool. Specifically, it is essential to clear the datasets from the media influence and ensure that the RSVs are not subject to systemic or occasional anomalies and the fluctuations are contained within the chosen acceptability range. Within a specific area (e.g., Italy), the intensity and frequency of these problems seem inversely proportional to the demographic size of the examined region. Other confounding factors (e.g., reliability and distribution of internet connections) can also affect outcomes.

### Strengths

This paper has several strengths. To the best of the authors’ knowledge, this is the most extensive search for COVID-19-related names in the literature. Indeed, we have identified a wide variety of terms associated with COVID-19 never examined in previous research. Such findings need to be considered in future infodemiological analyzes and infodemic management plans. Furthermore, we have proposed a new method to improve the sensitivity of Google Trends, accurately quantifying even RSVs below the unit. This allowed us to calculate the relative RSV ratios between all examined queries. Finally, we reported the negative results due to the anomalies of Google Trends as for the "Subregion" section, managing to circumvent these limitations by analyzing the interest over time of the individual regions.

### Limitations

Our approach also has limitations that need to be considered. In particular, the internet penetration of Italian users is about 76%; consequently, our results do not depict the attitudes of about 24% of the population [46]. We also have no way of investigating the reasons for such web searches. For example, it is plausible that at least a fraction of these queries were made for research purposes by a specific subset of the population (e.g., scientists). Therefore, our procedure is complementary and not a substitute for the textual analysis of social platforms. Finally, although our collection method has produced new results, we are not guaranteed to have included all relevant terms.

In conclusion, based on our findings, we underline the urgency of greater timeliness in assigning simple scientific names to new diseases and related variants. The procedural slowness can be compensated by using suitably trained deep learning algorithms. Indeed, recurrent neural networks like "long short term memory" (LSTM) have demonstrated great success in complex tasks such as text analysis, speech recognition, and names generation under very specific requests [47]. Finally, we support the use of Google Trends for infodemiological purposes and encourage the adoption of our new procedure to increase its sensitivity. At the same time, we recommend carefully evaluating the use of Google Trends for epidemiological investigations.

## Methods

### Procedure summary for COVID-19 names

Through the analysis of previous literature, the search for synonyms, and the suggestions of Google Trends, we have selected keywords to search on Google Trends to observe the web interest in Italy towards potential COVID-19 stigmatizing and generic names. The methodological details are provided in the subsection "Data collection". We used mean, median, percentage increases, and Welch and Mann-Whitney tests to compare the web interest in these keywords before and after the novel coronavirus outbreak in China (end of December 2019). The use of these measures is justified by an evident absence of trends and seasonalities before 2020 (excluding one exceptional case treated separately). In doing so, we estimated the impact of COVID-19 in adopting these terms. The methodological details are provided in the subsection "Statistical analysis". The trends of each keyword were analyzed from 1 January 2018 until 13 September 2021 to observe any peaks of interest in correspondence with specific events (e.g., lockdown announcement). Starting from 1 January 2020, we analyzed the trend of COVID-19 scientific names. We referred to the RSV peak of the most used word (i.e., "coronavirus") to estimate the ratios between scientific and infodemic names. Finally, we collected Google Trends regional data to investigate spatial-temporal patterns related to the adoption of scientific and infodemic terms ("Subregion" section). The analyses were conducted longitudinally, month by month, and cumulatively (i.e., iteratively from January 2020 until each month "January 2020 - September 2021"). To verify the significance of the RSV increase of each region, we compared the "Interest over time" RSVs of each region from January 2018 to September 2021 through the same "historical comparison" procedure exploited for national queries. To improve the accuracy of the RSVs, we used two new iterative-comparative approaches. The methodological details are provided in the subsections "Iterative procedure 1/2 to improve Google Trends accuracy on low RSV keywords."

### Procedure summary for COVID-19 VOCs names

We have selected the name of the COVID-19 variants of concern (VOCs) from the official website of the Istituto Superiore di Sanità. The details are reported in the "Data collection" subsection. From 1 December 2020, we examined the web searches of scientific and infodemic names attributed to COVID-19 VOCs. In addition to calculating the RSV ratio between COVID-19 VOCs infodemic and scientific names, we quantified the spread of stigmatizing names by calculating the RSV ratio with the keyword "covid" (i.e., the most used keyword to refer to COVID-19 in the period December 2020 - September 2021). The significance of the difference between RSV average values and medians was assessed through Welch and Mann-Whitney tests. The query "variante - alpha - alpha - beta - gamma - delta" was searched from January 2018 to September 2021 to confirm that the term only referred to COVID-19 VOCs generic or stigmatizing names. Since the RSV was stationary from 2018 to the end of 2020, we performed Welch and Mann-Whitney tests. Details are given in the "Statistical Analysis" subsection. We collected Google Trends regional data to investigate spatial-temporal patterns related to the adoption of scientific and infodemic VOCs names ("Subregion" section). The analyzes were conducted longitudinally, month by month, and cumulatively (i.e., iteratively from December 2020 until each month in the period "December 2020 - September 2021"). To verify the significance of the RSV increase of each region, we compared the "Interest over time" RSVs of each region from January 2018 to September 2021 through the same "historical comparison" procedure exploited for national queries. To improve the accuracy of the RSVs, we used two new iterative-comparative techniques. The methodological details are provided in the subsections "Iterative procedure 1/2 to improve Google Trends accuracy on low RSV keywords."

### Data collection

#### Keyword selection

The keywords to be analyzed with Google Trends were selected in different ways [32]. Firstly, we drew on the results of multiple papers to compensate for the limitations of single approaches (Table 4).

**Table 4:** Literature consulted for the selection of COVID-19 names

Authors	Selection method
Chandra et al. [48]	Sentiment analysis of tweets via deep learning
Gallotti et al. [49]	Manual selection of official names, early tentatives, and geographical-related names
Islam et al. [50]	Manual review of social media, mass media, and fact-checking agency websites
Rovetta et al. [3]	Manual search of names commonly used by media and scientific articles

Secondly, we consulted the Italian "Treccani" dictionary [51] to find synonyms of the more generic terms (e.g., virus). Finally, an independent search was carried out using Google Trends’ suggestions (related queries and topics). The COVID-19 VOC names were collected from the official website of the Istituto Superiore di Sanità [16].

#### Google Trends national data

Infodemic and scientific keywords selected through the previous procedure (Tables 1, 3) were searched on Google Trends in the "All categories" section. Other terms such as "cina" (China) and "cinese" (Chinese), investigated to encompass all of the keywords stigmatizing China, were searched under the "health" category. The goodness of this procedure was confirmed by the tests carried out (see Results section) and the 25 related queries proposed by Google (all related to COVID-19 despite the investigated period from January 2018 to September 2021). Interest over time data was downloaded in ".csv" format and analyzed using Microsoft Excel 365. The examined periods ranged from 1 January 2018 to 13 September 2021 for generic and stigmatizing names and from 1 January 2020 to 13 September 2021 for scientific names (which did not exist before 2020). Therefore, the observed RSVs were weekly cumulative [52]. The investigation period for COVID-19 VOC names went from the appearance of the first variant (December 2020) to September 2021 [53]. All VOC-related keywords were searched under the "all" category; the exact list is given in the supplementary file (Table S2).

#### Google Trends regional data

Since the Google Trends subregion section allows the user to compare regional cumulative RSVs only one keyword at a time, we used the keywords "cina" and "cinese" to examine the use of stigmatizing COVID-19 names ("health" category). The reasons for selecting these two terms are explained in the previous subsection. In some cases, after consulting the top related queries, we have removed specific terms not related to COVID-19 (e.g., "rocket"); the "-" operator was exploited to do this. Table 1 infodemic keywords and Table 3 scientific keywords were also searched individually from 1 January 2018 to 13 September 2021 and 1 January 2020 to 13 September 2021, respectively ("all" category). Subregional data was downloaded in ".csv" format and analyzed using Microsoft Excel 365. The same collecting procedure was repeated for the interest over time of every single region. Finally, the "subregion" and "interest over time" data were downloaded - through the above procedures - for the COVID-19 VOC names from December 2020 to September 2021 ("all" category).

#### Google Trends error assessment

As Google Trends approximates RSVs to the nearest integer, we considered a sensitivity error of 0.5 for each RSV. Since previous studies reported unexpected fluctuations and anomalies in RSVs depending on the collection day, all data was gathered for at least seven consecutive days [37]. In this way, we checked which datasets were stable enough and excluded unstable ones. Methodological details are provided in the subsection "Statistical analysis" (Google Trends stability assessment).

### Statistical analysis

#### Percentages

We used the percentage increase ∆ to calculate the difference between two descriptive statistics of two consecutive series. When this was impossible because the starting value was zero, we used the percentage difference δ (this was specified in the Results section). δ was also used to estimate the difference between two concurrent measures. Finally, we used percentage ratios to calculate the weight of one measure over the other. In this case, we have provided a 95% confidence interval (95% CI). The standard error was calculated through the standard propagation of uncertainty.

#### Means and medians comparison

The Welch t-test was used to evaluate the statistical significance of the difference between two mean values. We adopted this measure for its robustness when dealing with strongly skewed distributions and the implications of the central limit theorem [54]. Since degrees of freedom were always greater than 90.5, the difference between the t and z values was considered negligible [55]. The magnitude of the difference was calculated through the percentage increase. Through Shapiro-Wilk tests and graphical analyses of Q-Q plots and frequency histograms, we observed that the distributions of COVID-19 names were generally not Gaussian. However, their shape was similar (left-skewed). For this reason, we also implemented the Mann-Whitney U test and calculated the percentage increase between the medians. When the initial value of the median was zero, we used the percentage difference instead of the percentage increment. In Table 2, we have reported all the measures used; in the rest of the manuscript, to simplify the reading, we have reported only the differences between mean values and the t-values. However, we also verified the validity of the results through the Mann-Whitney U test. In this regard, we point out that t-test and U-test never gave conflicting results. Mean values were preferred to medians since they relate to the total RSV (including outliers), i.e., the global resonance of a keyword.

#### Seasonality test

The Seasonality of the time series was first evaluated graphically. Then, we used the Correlogram data analysis tool provided by Real Statistics for Microsoft Excel 365 [56], setting a maximum lag of 53 weeks (approximately one year). We left the remaining parameters to the default ones. We did not find seasonalities in our dataset except for the word "influenza" (flu), which was analyzed separately.

#### Stationarity test

The stationarity of the time series was first evaluated graphically. Then, we ran the Augmented Dickey-Fuller test (ADF). The Schwert criterion was exploited to select the optimal lag. The "drifts" box has been checked. We left the remaining parameters to the default ones. All datasets were sufficiently stationary where required.

#### Time series historical comparisons from January 2018 to September 2021

We have organized the weekly RSV data of each keyword into a matrix h_ij_. Each column represents the RSV series of a specific keyword. All series turned out to be stationary (∀P_ADF_ < .01) and free of seasonalities before January 2020 (no regular scalloped patterns have been identified in the time series nor the correlogram). Every h_ij_ varied in the range [0, 2] except for the "corona" keyword, which had an isolated peak (RSV = 17) in October 2018 (this outlier has been removed as we are not interested in occasional spikes before COVID-19). Given the characteristics of the time series before COVID-19, we considered it superfluous to implement predictive models to estimate the trend in the period 2020-2021. The comparison between January 2018 - December 2019 and January 2020 - September 2021 was conducted applying the following measures: percentage increase between means and medians, Welch t-test, and Mann-Whitney U test z-score. The results are shown in Table 2. The same procedure was also carried out for the query "variante - alpha - alpha - beta - gamma - delta." The periods compared were January 2018 - November 2020 and December 2020 - September 2021. In particular, the first period was stationary and devoid of seasonalities and outliers.

#### Google Trends datasets’ stability

We have collected the RSVs of each keyword for at least seven consecutive days (collection time: from 10 to 12 am). All the search properties, namely keyword, region, period, and category, remained unchanged in each repetition. This served to observe any dependencies of RSVs on the collection day [37]. While national data proved stable, all regional datasets in the "subregion" section were found to be unreliable due to incompleteness (e.g., systematic absence of 5 or more regions in the dataset), anomalies (e.g., occasional disappearance of regions from the dataset on specific collection days), and excessive compatibility of the RSVs of each region. In particular, the compatibility between the RSVs of each region was measured as follows: for each region, we verified the distributive normality of the RSV oscillations thanks to the Shapiro-Wilk test and Q-Q plots plus frequency histograms visualization. Mean, standard deviation, and standard error of the RSVs collected daily were calculated. After that, a multiple Welch t-test was performed between all the mean values. Theoretically speaking, to not increase the number of "false positives", the use of appropriate corrections could be required by some authors (e.g., Bonferroni). However, we exploited that penalties such as Bonferroni’s can increase false negatives but not false positives to prove that our results had a low degree of significance. More detailed information is provided in the "Results" section, subsection "Negative results."

### Iterative procedure 1 to improve Google Trends accuracy on low RSV keywords

#### Example

Let’s suppose we have a set of 4 keywords on Google Trends. Their RSV peaks are 100, 50, 20, 2, respectively. The ratios between consecutive peaks are: 100/50 = 2, 50/20 = 2.5, and 20/2 = 10. However, many RSVs of keyword 4 are < 1 or 0. Excluding the first keyword, Google Trends renormalizes the results according to the following transformation, which preserves the relative ratios between the peaks: 50 100, 20 40, and 2 4 (indeed, 100/40 = 50/20 = 2.5, and 40/4 = 20/2 = 10). By doing so, the RSVs of keyword 4 (that Google Trends reported as < 1 or 0 in the first dataset) grow until they are quantifiable. The procedure can be repeated at will, finding keywords that have intermediate peaks. If the datasets’ peaks are too far from each other, it is possible to add an off-topic keyword with an RSV peak suitable for this task. At each step, it is necessary to download the data in.csv format. Thus, for comparing the original dataset with the last one, it is sufficient to scale all RSVs of the original dataset proportionally. For example, in the case shown here, the rescaled peaks will be 200, 100, 40, and 4 (indeed, ratios between consecutive peaks are the same as the original dataset). The same reasoning applies to all the other RSVs that don’t constitute peaks (i.e., in this case, it will be enough to multiply by 2 all the RSVs of keyword 1).

#### General case

Suppose we have *n* keywords *K*_*i*_ to compare ("Interest over time" section). Since Google Trends allows the inclusion of up to five keywords, if *n* ≤ *5*, the comparison can be immediate, while, if *n* > *5*, we need to compare the keywords by *m(j*=*1)* subgroups *S*_*j*_ (with *m(j)* ≥ *m(j* + *1)* and *j ∈ {1, ... , Floor(n/5)* + *1* = *m(1)}*). We specify that the dependence of *m* on *j* is because the number of subgroups can decrease as we exclude keywords containing peaks *RSV* = *100*. One among these subgroups will be formed by five keywords, while the remaining will be formed by four or fewer. Calling M1 the keyword with the highest peak in the subgroup *S*_*1*_ of five elements, it will be sufficient to include *M*_*1*_ in *S*_*2*_ and select *M*_*2*_, and so on until obtaining *M*_*m*_*(1)* in *S*_*m*_*(1)*. Thus, *M*_*m*_*(1)* will be the keyword with the highest peak in the whole sample. For convenience, we set *M*_*m*_*(i)* = *K*_*i*_. At each iterative step, it is necessary to download the data in ".csv" format. Since *K*_*1*_ was assigned, the sample size to be analyzed has decreased to *n - 1*. Calling the acceptability measure (whatever it is) with *α*, we set an acceptability threshold α* for *RSVs* (e.g., keywords that have *RSV* ≥ 2 are acceptable). If with a stroke of luck, *M*_*m*_*(1)* was extracted from the first subgroup *S*_*1*_, we already have all the necessary comparisons to understand which keywords have α ≥ α* and which ones need to be improved. If *M*_*m*_*(1)* was extracted from a *S*_*k*_ subgroup with *k* > *1*, we need to reintroduce the other *k - 1* subgroups of four keywords and *M*_*m*_*(1)* in Google Trends and download the ".csv" files. Finally, we have the keywords sorted from *1* to *n* in descending order of *RSV* peak. We call this set *A*_*1*_. Now suppose that *t(l* = *1)* subgroups *s*_*l*_ (with *t(l)* > *t(l* + *1)* and *l ∈ {1, ... , t(1)* < *m(1)*) do not match α*. The keyword to be taken as a new reference must be the keyword with the lowest acceptable peak within *A*_*1*_. All the above procedures must be repeated until α* is respected for all keywords. If the keywords of interest have too distant peaks (e.g., 100 and 1), it is recommended to introduce an external keyword with an intermediate peak (e.g., 40) to carry out the analysis. Such a keyword can be discarded at the end of the iteration. Assuming that the operation is performed *h* times, we will have *h* sets *A*_*q*_ (with *h* < *m(1)* and *q ∈ {1, ... , h}* ). At each step *q*, the keyword inside *A*_*q*_ with the least acceptable *RSV* peak (*p*_*q*_) will be the keyword with *RSV* = *100* in *A*_*q*+*1*_. Therefore, all *RSVs* of all keywords in the *A*_*h−1*_ set will be multiplied by *100/p*_*h−1*_. Then, all the RSVs of all the keywords of the *A*_*h−2*_ set will be multiplied by *100/p*_*h−2*_* · 100/p*_*h−1*_, and so on up to *A*_*1*_. In conclusion, the *RSV* of each keyword in each group *A*_*q*_ < *h* must be multiplied by a factor *100 *^*h−q*^* ∏ *^*h−q*^_*u*=*1*_* (p*_*h−u*_*)*^*−1*^.

#### Our case

This procedure allows comparing measures such as mean and median of keywords with very distant RSVs with the required precision. In our case, this was used to quantify the relative ratio between the average use of infodemic and non-infodemic COVID-19 names. The iteration continued until at least 50% of the series had RSVs > 1.

### Iterative procedure 2 to improve Google Trends accuracy on low RSV keywords

#### Example

Suppose we need to compare the RSV of a keyword in two consecutive periods. Suppose that the RSVs in the first period are 1, 1, 1, 1, 1, 2. Let’s also assume that the peak RSV = 100 is in the second period. By isolating the first period (i.e., excluding the peak RSV = 100), we obtain the following RSVs: 39, 42, 54, 44, 59, 100. Then, it is possible to recalibrate the previous RSVs based on the more precise data from the second dataset. In particular, if we multiply each data of the second dataset by 2/100 (i.e., the maximum value of the first dataset in period 1 over 100), we get 0.78, 0.84, 1.08, 0.88, 1.18, 2. In this way, the oscillations visible in the second series are now also present in the first. Since Google Trends has margins of imprecision, it is advisable to look for that multiplicative factor that minimizes the absolute value of the differences between the final and the original values.

#### General case

Suppose we need to compare the RSV of a keyword in two consecutive periods and that the RSVs of one of these two periods are too low to show significant fluctuations. We call the datasets relating to these two periods D1 and D2. As demonstrated in the previous subsection, excluding the period that contains the RSV peaks (D2), it is possible to observe these fluctuations thanks to Google Trends renormalization. We call this new dataset D3. Considering each di ∈ D1 and di’ ∈ D3 , we can find the multiplicative constant k such that k · di’ ~ di. This procedure allows the fluctuations of D3 to be added to D1. Specifically, it is advisable to start from the maximum RSV in D1 (d*) divided by 100 (i.e., k = d*/100) and add small increments h > 0 to k until minimizing ∑n i=1 |(k ± h) di’ - di|. Calling D4 the dataset {(k ± h) di’} , we can now replace D1 with D4. It is possible to perform a Welch t-test and calculate the percentage difference between mean and median values to evaluate if the procedure has failed and the difference between the original dataset (D1) and the transformed one (D4) is substantial.

#### Warning

When excluding a timelapse, Google Trends may change the type of RSV (e.g., from weekly to daily) [51]. Ensure that this does not happen, or the datasets will be incomparable.

## Supplementary Information

**Additional file 1.**

## Data Availability

All data generated or analysed during this study are included in this published article [and its supplementary information files].
