# Supplementary material for: A new infodemiological approach through Google Trends: longitudinal analysis of COVID-19 scientific and infodemic names in Italy
Source: BMC Med Res Methodol. 2022 Jan 30;22:33. doi: 10.1186/s12874-022-01523-x (PMC8801192; doi:10.1186/s12874-022-01523-x)
Supplement: Supplementary file 1 — Additional file 1. [file 12874_2022_1523_MOESM1_ESM.pdf]

Keywords with positive RSV

coronavirus
corona
virus cina
corona virus
virus cina + virus cinese + virus wuhan
coronavirus cina + coronavirus cinese
corona cina + corona cinese + corona wuhan
sars + sarscov + sars-cov
malattia
malattia cina + malattia cinese + malattia wuhan
epidemia
pandemia
pandemia cina + pandemia cinese + pandemia wuhan
contagio
influenza
influenza cinese + influenza cina + influenza wuhan
patologia

Excluded keywords with too low RSV (always 0)

infezione cinese + infezione cina
epidemia cina + epidemia cinese + epidemia wuhan
contagio cina + contagio cinese + contagio wuhan
patologia cina + patologia cinese + patologia wuhan

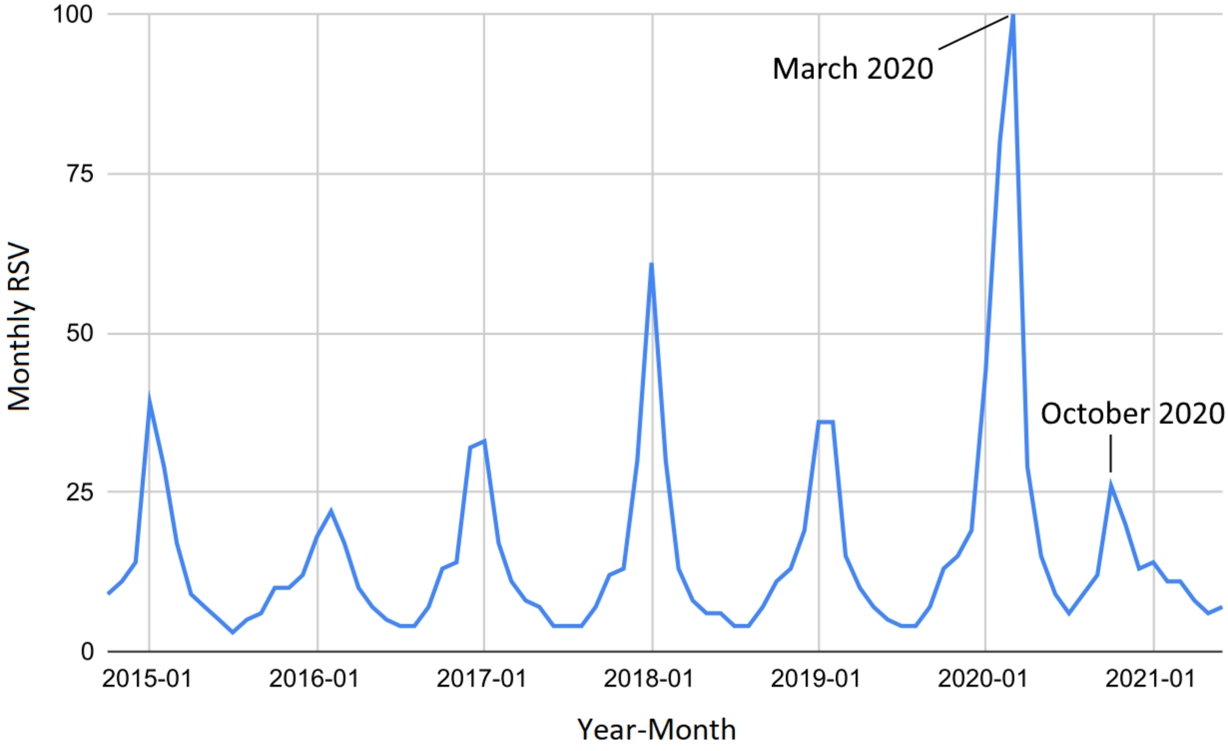

Figure S1. "Influenza" (flu) RSV from late 2014 to July 2021.

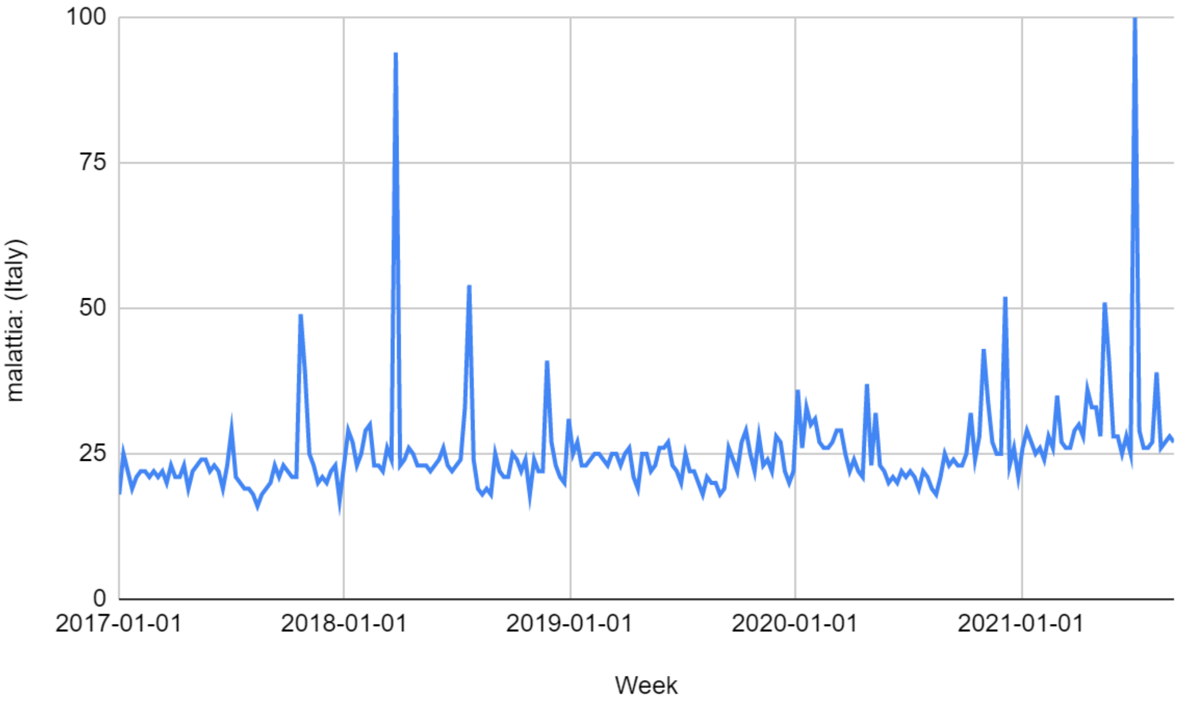

Figure S2. “Malattia” keyword trend from 2017 to 2021.

Considering the entire time series and subseries examined from January 2017 to September 2021, the ADF test detected some degree of stationarity (P<.01). However, from October 2020 onwards, there appears to be a moderate level shift. Indeed, Mann-Kendall + Sen's Slope test reveals a slight upward trend (SS=0.023, 95%CI [0.018, 0.029], P<.001). By re-running the test from January 2017 to December 2019, we obtained a slower trend (SS=0.013, 95%CI [0.000, 0.024], P=.006). Using as standard errors the difference between the maximum values of the confidence intervals and the SS values (i.e., given the slight skewness to the right of the distributions, an underestimate), we performed the Welch t-test obtaining a low significance (t=0.8). To analyze the nature of the trend, we performed the calculation y(i+1) - y(i), obtaining Figure S2. Since the graph obtained is markedly stationary (SS=0.000, 95%CI [-0.008, 0.000], P=.45), if a trend exists, it is linear. Therefore, although the Welch t-test between the two subseries is equal to t = 2.4, the impact of COVID-19 on the use of the name "malattia" is uncertain.

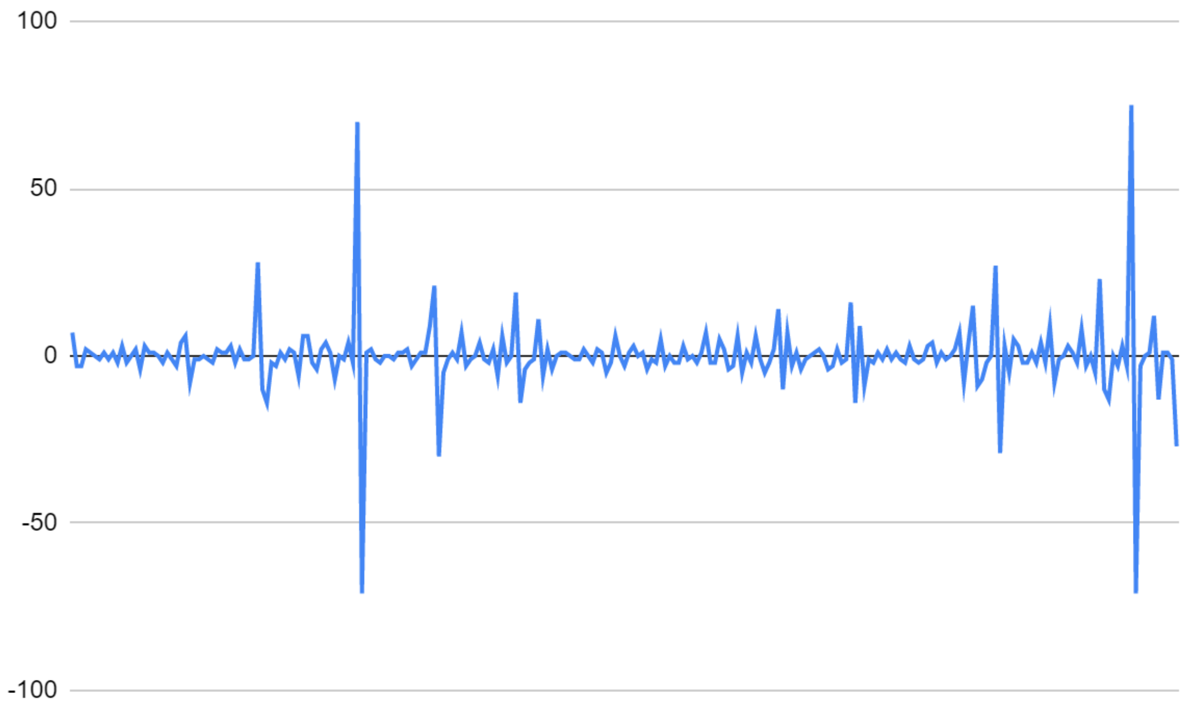

Figure S3. “Malattia” keyword y(i+1) - y(i) trend from 2017 to 2021.

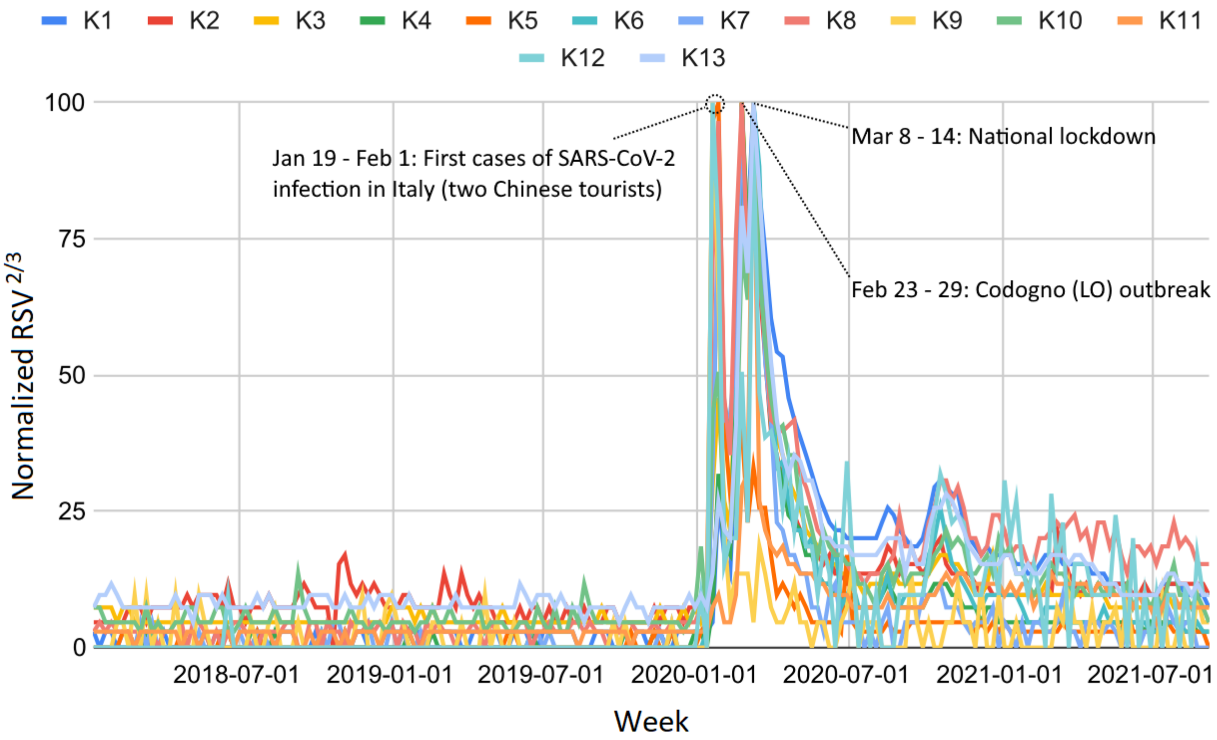

Figure S4. Trend of web interest in COVID-19 generic and stigmatizing keywords from 2018 to 2021. The values shown on the y-axis are RSV^(2/3) renormalized to 100. Each time series was normalized with respect to its own peak; therefore, this graph only shows the relative increase.

	Scientific name query	Infodemic name query
**Alpha variant**	variante alfa + variante alpha	variante inghilterra + variante inglese
**Beta variant**	variante beta	variante africa + variante sudafrica + variante africana + variante sudafricana
**Gamma variant**	variante gamma	variante brasile + variante brasiliana
**Delta variant**	variante delta	variante indiana + variante india

Table S1. COVID-19 variants of concern: exact Google Trends queries.

Name	Keyword	Anomaly	CV% Range
K1	coronavirus***	3 / 95%	[0, 4]
K2	corona*	3 / 75%	[0, 7]
K3	virus**	3 / 100%	[1, 7]
K4	corona virus	3 / 90%	[0, 8]
K5	virus cina + virus cinese + virus wuhan**	1 / 85%	N.A.
K6	coronavirus cina + coronavirus cinese + coronavirus wuhan	1 / 55%	N.A.
K7	corona cina + corona cinese + corona wuhan*	1 / 100%	N.A.
K8	sars + sarscov + sars-cov	3 / 100%	[3, 16]
K9	malattia cina + malattia cinese + malattia wuhan	1 / 100%	N.A.
K10	epidemia	3 / 100%	[9, 30]
K11	pandemia	3 / 90%	[0, 20]
K12	pandemia cina + pandemia cinese + pandemia wuhan	1 / 95%	N.A.
K13	contagio	3 / 100%	[1, 11]
K14	influenza cinese + influenza cina + influenza wuhan	1 / 100%	N.A.

Table S2. RSV anomalies in Google Trends. The third column shows the type of anomaly and the affected percentage of the dataset. The last column shows the range of the coefficient of variation. Translations: Cina = China, Cinese = Chinese, contagio = contagion, epidemia = epidemic, malattia = disease, pandemia = pandemic. Anomaly 1 = absence of regions. Anomaly 2 = occasional disappearance of regions. Anomaly 3 = high variance. * = the term "virus" was subtracted. ** = the term "corona" was subtracted. *** the terms "novel" and "nuovo" were subtracted.
